# Plasma Homocysteine Level Is Independently Associated With Conventional Atherogenic Lipid Profile and Remnant Cholesterol in Adults

**DOI:** 10.3389/fcvm.2022.898305

**Published:** 2022-06-13

**Authors:** Liyuan Zhou, Jia Liu, Yu An, Ying Wang, Guang Wang

**Affiliations:** ^1^Department of Endocrinology, Beijing Chao-Yang Hospital, Capital Medical University, Beijing, China; ^2^Medical Examination Center, Beijing Chao-Yang Hospital, Capital Medical University, Beijing, China

**Keywords:** homocysteine, high-density lipoprotein cholesterol, apolipoprotein A1, triglyceride, remnant cholesterol, Chinese adults

## Abstract

**Background:**

Homocysteine (Hcy) is an independent risk factor for cardiovascular disease, while mechanisms are unclear. Despite inconsistent and limited, epidemiological and experimental studies indicated that hyperhomocysteinemia (HHcy) affected lipid metabolism. This study aims to investigate the association of plasma Hcy with traditional lipid profiles and remnant cholesterol (RC) in Chinese adults.

**Methods:**

In total, 7,898 subjects aged 20–79 years who underwent a physical examination at Beijing Chao-Yang Hospital in Beijing were included in this study. Fasting plasma total cholesterol (TC), triglyceride (TG), high-density lipoprotein cholesterol (HDL-C), low-density lipoprotein cholesterol (LDL-C), apolipoprotein A1 (ApoA1), apolipoprotein B (ApoB), lipoprotein (a) [Lp(a)], Hcy, and other metabolic risk factors were measured by routine automated laboratory methods. RC was calculated as TC minus HDL-C and LDL-C. The linear regression model and logistic regression model were used to assess the relationship between Hcy and lipids after adjusting potential confounders.

**Results:**

Of the subjects, the median level of plasma Hcy was 13.0 μmol/L and 32.3% had HHcy. Plasma Hcy was negatively associated with HDL-C, ApoA1, and Lp(a) and positively associated with TG levels after adjusting age, sex, body mass index, blood pressure, alanine transaminase, aspartate transaminase, creatinine, uric acid, and glucose. HHcy significantly increased the risk of low HDL-C [odds ratio (OR) 1.26; 95%CI (1.11–1.44); *p* < 0.001]. The net mediation effects of ApoA1 on the relationship between Hcy and HDL-C before and after adjusting confounders were 46.9 and 30.6%, respectively. More interestingly, the RC level was significantly elevated in subjects with HHcy after adjusting other influencing factors (*p* = 0.025). Hcy presented a positive correlation with RC levels after adjusting the above confounding factors (β = 0.073, *p* = 0.004), and the correlation was still significant even after controlling other lipids, including TG, LDL-C, HDL-C, ApoA1, ApoB, and Lp(a).

**Conclusion:**

Our study showed that plasma Hcy was not only significantly associated with conventional atherogenic lipids but also independently correlated with RC levels beyond other lipids after controlling potential confounders. This finding proposes that identifying Hcy-related dyslipidemia risk, both traditional lipids and RC residual risk, is clinically relevant as we usher in a new era of targeting Hcy-lowering therapies to fight against dyslipidemia or even cardiovascular disease.

## Introduction

Atherosclerotic cardiovascular disease (ASCVD) is a leading cause of death worldwide, thus early prevention and treatment are extremely important. In addition to obesity, hyperglycemia, hypertension, and dyslipidemia, it has been confirmed that homocysteine (Hcy) is an emerging and independent risk factor for cardiovascular diseases, including ischemic heart disease, stroke, and peripheral vascular disease (1, 2). The meta-analysis suggested that patients with heart failure and ischemic stroke had significantly elevated plasma Hcy levels compared with normal controls (3, 4). It was estimated by Boushey et al. that approximately 10% of coronary artery disease might be attributed to hyperhomocysteinemia (HHcy) (5). Inversely, B vitamins supplementation, a therapy to lower plasma Hcy levels, appeared to decrease cardiovascular events in subjects with normal renal function (6).

The mechanism linking HHcy to the risk of ASCVD is still unclear. Hcy is a type of thiol-containing amino acid and is one of the critical intermediates of the methionine cycle and cysteine metabolism (7). Previous epidemiological studies and animal experiments implicated that inflammation reaction, oxidative stress, endothelial dysfunction, endoplasmic reticulum (ER) stress, and epigenetic control of gene expression were all potential mediators of HHcy-induced ASCVD (8–10). Additionally, it was also shown that HHcy increased the biosynthesis and secretion of cholesterol and triglyceride (TG) and suppressed the protein synthesis of apolipoprotein A1 (ApoA1), which is the main apolipoprotein of high-density lipoprotein cholesterol (HDL-C), and thus decreased HDL-C levels in animal and cellular experiments (11, 12). Moreover, Julve et al. demonstrated that the antioxidant function of HDL-C was impaired in HHcy mice induced by methionine (13). Nevertheless, the link between HHcy and lipid profiles in clinical and epidemiological studies was controversial. Some studies showed that Hcy had a positive correlation with low-density lipoprotein cholesterol (LDL-C), TG, and cholesterol and a negative correlation with HDL-C (7, 14), while others suggested that HHcy was not associated with atherogenic dyslipidemia after adjusting potential confounders (15), which might be attributable to the differences in demographic characteristics, including age, sex, race, and geographic regions, food intake, physical activity, and confounding factors included in data analyses among the studies.

With the widely used LDL-C-lowing therapy *via* statin, residual cardiovascular risk got more attention even among subjects with low LDL-C levels (16). Other than the conventional plasma lipid profile, triglyceride-rich lipoproteins (TGRL) or remnant cholesterol (RC) was also shown to be associated with the development of ASCVD (17, 18). Castañer et al. reported that RC, not LDL-C, was related with cardiovascular outcomes in overweight or obese individuals (19). Mendelian randomized studies suggested a causal relationship between the cholesterol content rather than TG of remnant particles and ischemic heart disease (20–22). Another study showed that RC-induced ASCVD was proportional to apolipoprotein B (ApoB) concentrations (23). A recently published primary prevention study demonstrated that elevated RC levels can predict ASCVD independent of traditional risk factors, including LDL-C and ApoB levels (24). Therefore, the specific content of RC increasing the ASCVD risk was unclear. Moreover, evidence linking Hcy, RC, and ASCVD was limited. Only one epidemiological study explored the relationship between HHcy and RC in US adults and did not support the association after controlling potential confounders (15).

In this study, in addition to investigating whether plasma Hcy is independently associated with conventional lipid profiles after adjusting confounders, we also focused on the relationship between Hcy and RC levels and determined whether the relationship exists beyond other lipids. We sought to add Chinese evidence for a better understanding of the link between Hcy and lipids and provide insight into the prevention and treatment of dyslipidemia and associated ASCVD in individuals with HHcy.

## Materials and Methods

### Study Population

This is a single-center cross-sectional study in Beijing, North China. We collected data from the routine health check-up of subjects aged 20–79 years at the Medical Examination Center of Beijing Chao-Yang Hospital from April 2016 to August 2021. All participants underwent anthropometric, plasma Hcy, and biochemistry parameter measurements, and medical histories were also recorded. Individuals with the following conditions were excluded: pregnant or breastfeeding women; severe hepatic dysfunction with alanine transaminase (ALT) or aspartate transaminase (AST) exceeding three times the upper limit of the normal range; severe renal dysfunction, which was defined as creatinine (Cr) exceeding two times the upper limit of the normal range; using antihypertensive, glucose-lowering, and lipid-lowering medications; and with missing covariates. Finally, 7,898 participants were included in the data analyses. This study was approved by the Ethics Committee of the Beijing Chao-Yang Hospital affiliated with Capital Medical University. Written informed consent was obtained from each subject.

### Clinical Parameter Measurements

All subjects underwent anthropometric measurements, including age, sex, height, body weight, and blood pressure, by certified doctors. Standing height and body weight were assessed by a wall-mounted stadiometer to the nearest 0.1 cm and 0.1 kg. Systolic blood pressure (SBP) and diastolic blood pressure (DBP) were measured three times consecutively after a 5 min rest using a standard sphygmomanometer. The average values of the three recorded blood pressure were used for data analyses. Body mass index (BMI) was calculated as body weight divided by height squared (kg/m^2^).

After at least 10 h of overnight fasting, peripheral venous samples were collected for laboratory tests. Fasting blood glucose (FBG), total cholesterol (TC), TG, HDL-C, ApoA1, ApoB, lipoprotein (a) [Lp(a)], ALT, AST, Cr, urea, uric acid (UA), and Hcy were measured by routine automated laboratory methods [(Hitachi 7060C automatic biochemistry analysis system) in Beijing Chao-Yang Hospital. Hemoglobin A1c (HbA1c) was assessed by high-performance liquid chromatography. LDL-C was calculated by the Friedewald formula. Non-HDL-C was calculated as TC minus HDL-C, and RC was defined as non-HDL-C minus LDL-C (22). The estimated glomerular filtration rate (eGFR) was calculated by the Cockcroft-Gaulu equation: eGFR = [140-age (years)] × body weight (kg) × 1.23/Cr (μmol/L) (× 0.85 if female). The powerful surrogate marker for fatty liver disease, Zhejiang University (ZJU) index, was calculated by BMI (kg/m^2^) + FBG (mmol/L)+ TG (mmol/L)+ 3 × ALT (U/L)/AST (U/L) (+2 if female) (25). The fibrosis-4 (FIB4) index was defined as: age (years) × AST (U/L)/(platelet count (10^9^/L) × √ ALT (U/L)] (26).

### Definitions

For this study, the definition was in accordance with the international standard: lean (BMI < 25 kg/m^2^), overweight (BMI ≥ 25 kg/m^2^), obesity (BMI ≥ 30 kg/m^2^) (27), hypertension (SBP ≥ 140 mmHg or DBP ≥ 90 mmHg) (28), hyperglycemia (FBG ≥ 5.6 mmol/L) (29). The atherogenic lipid profiles were in accordance with the criteria of China Adult Dyslipidemia Prevention Guide (2007): hypercholesterolemia (TC ≥ 5.18 mmol/L), hypertriglyceridemia (TG ≥ 1.7 mmol/L), low HDL-C (HDL-C < 1.04 mmol/L), and high LDL-C (LDL-C ≥ 3.37 mmol/L). Consistent with published studies, Hcy ≥ 15 μmol/L was defined as HHcy (7, 30).

### Statistical Analysis

**FIGURE 1:**
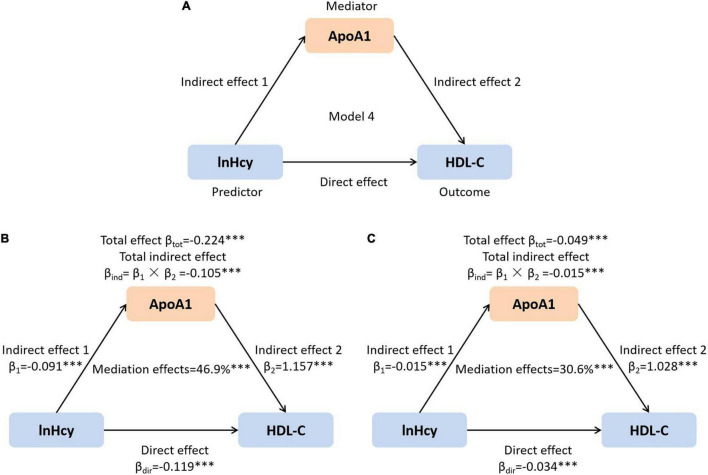
The mediation role of ApoA1 in the association of lnHcy with HDL-C. **(A)** Mediation model; **(B)** unadjusted; and **(C)** adjusted for age, sex, BMI, SBP, DBP, ALT, AST, Cr, UA, and glucose. ^***^p < 0.001. ApoA1, apolipoprotein A1; Hcy, homocysteine; HDL-C, high-density lipoprotein cholesterol; BMI, body mass index; SBP, systolic blood pressure; DBP, diastolic blood pressure; ALT, alanine aminotransferase; AST, aspartate aminotransferase; Cr, creatinine; UA, uric acid.

The normality of the data was assessed using the Kolmogorov–Smirnov test. Normally distributed continuous parameters were expressed as mean ± standard deviation (*SD*) and were compared using Student’s *t*-tests. Non-normally distributed continuous indices, such as Hcy, ALT, AST, TG, RC, Lp(a), glucose, and HbA1c, were expressed as median and interquartile range [median (IQR25–75)] or median ± 95%CI and were compared using Student’s *t*-tests after natural log-transformed. A general linear model was used to compare data between groups after adjusting for potential confounding factors. Categorical variables were compared using χ^2^ tests. When performing correlation and regression analysis, non-normally distributed continuous variables were naturally log-transformed. Partial correlation coefficients were used to explore the relationship between RC and other plasma lipids after adjusting confounding factors. A linear regression model was used to assess the relationship between Hcy and plasma lipids. To further evaluate the potential associations of HHcy with atherogenic dyslipidemia, a logistic regression model was performed. A mediation analysis was performed using the PROCESS Procedure for SPSS Release 3.3 for HDL-C levels before and after adjustment. Animal and cellular studies showed that Hcy suppressed the protein synthesis of ApoA1, which is the main apolipoprotein of HDL-C and decreased HDL-C levels (11, 12). Therefore, we speculated that Hcy similarly influenced HDL-C levels through regulating ApoA1 in humans and detected whether ApoA1 plays a role in mediating the relationship between Hcy and HDL-C levels by using template Model 4 (Figure 1A). A two-tailed *p*<0.05 was considered statistically significant. SPSS 22.0 (Chicago, IL, United States) and Graphpad Prism 9.0 (San Diego, CA, United States) were used for the statistical analysis.

## Results

### Demographic and Laboratory Characteristics of Study Subjects

A total number of 7,898 subjects with an average age of 41.9 years were finally included in this study. Of the subjects, 4,154 (52.6%) were males. Compared with females, males had significantly higher age, BMI, SBP, DBP, ALT, AST, ZJU index, Cr, urea, UA, LDL-C, non-HDL-C, TG, ApoB, glucose, HbA1c, and prevalence of hyperglycemia and hypertension, whereas lower eGFR, HDL-C, and ApoA1 levels (all *p* < 0.001). The median level of plasma Hcy was 13.0 μmol/L in the whole population, which was also higher in males than females (*p* < 0.001) (Table 1).

**TABLE 1:** Clinical characteristics of subjects.

Parameters	Total	Female	Male	*p*
N	7,898	3,744	4,154	N/A
Age (years)	41.9 ± 13.0	41.3 ± 13.3	42.5 ± 12.7	<0.001
BMI (kg/m^2^)	24.3 ± 3.9	22.8 ± 3.6	25.7 ± 3.6	<0.001
SBP (mmHg)	122.1 ± 17.5	117.0 ± 17.8	126.7 ± 15.8	<0.001
DBP (mmHg)	72.5 ± 11.7	68.8 ± 10.8	76.1 ± 11.3	<0.001
ALT (U/L)*	19.0 (14.0–28.0)	16.0 (12.0–20.0)	24.0 (18.0–34.0)	<0.001
AST (U/L)*	21.0 (18.0–25.0)	20.0 (17.0–23.0)	22.0 (20.0–27.0)	<0.001
ZJU	33.9 ± 5.6	31.4 ± 4.7	36.2 ± 5.3	<0.001
FIB4*	0.78 (0.57–1.11)	0.76 (0.56–1.11)	0.80 (0.57–1.12)	0.096
Cr (μmol/L)	64.9 ± 13.6	54.6 ± 8.0	74.1 ± 10.8	<0.001
Urea (mmol/L)	5.1 ± 1.3	4.7 ± 1.2	5.4 ± 1.2	<0.001
eGFR [mL/(min⋅1.73 m^2^)]	111.0 ± 26.8	113.5 ± 25.9	108.6 ± 27.3	<0.001
UA (μmol/L)	348.5 ± 93.4	290.0 ± 63.8	401.2 ± 84.0	<0.001
TC (mmol/L)	4.94 ± 0.95	4.94 ± 0.95	4.94 ± 0.94	0.804
HDL-C (mmol/L)	1.33 ± 0.35	1.50 ± 0.35	1.18 ± 0.27	<0.001
LDL-C (mmol/L)	2.99 ± 0.86	2.89 ± 0.85	3.08 ± 0.85	<0.001
Non-HDL-C (mmol/L)	3.61 ± 0.93	3.44 ± 0.92	3.76 ± 0.92	<0.001
TG (mmol/L)*	1.21 (0.85–1.80)	0.99 (0.75–1.41)	1.45 (1.02–2.11)	<0.001
ApoA1 (mmol/L)	1.40 ± 0.22	1.47 ± 0.22	1.34 ± 0.20	<0.001
ApoB (mmol/L)	0.84 ± 0.21	0.80 ± 0.21	0.87 ± 0.20	<0.001
Lp(a) (mg/dL)*	11.8 (6.8–23.6)	13.5 (7.8–26.5)	10.6 (6.1–20.7)	<0.001
Glucose (mmol/L)*	4.81 (4.46–5.22)	4.73 (4.41–5.11)	4.90 (4.51–5.33)	<0.001
HbA1c (%)*	5.4 (5.2–5.7)	5.4 (5.2–5.7)	5.5 (5.3–5.8)	<0.001
Hcy (μ/L)*	13.0 (10.0–16.0)	11.0 (9.0–13.0)	15.0 (12.0–19.0)	<0.001
Hyperglycemia, *n* (%)	1,055 (13.4%)	328 (8.8%)	727 (17.5%)	<0.001
Hypertension, *n* (%)	1,354 (17.1%)	445 (11.9%)	909 (21.9%)	<0.001

*Non-normally distributed continuous variables were compared after natural log-transformed. Data were analyzed using Student’s t-test. BMI, body mass index; SBP, systolic blood pressure; DBP, diastolic blood pressure; ALT, alanine aminotransferase; AST, aspartate aminotransferase; ZJU, Zhejiang University; FIB4, fibrosis-4; Cr, creatinine; eGFR, the estimated glomerular filtration rate; UA, uric acid; TC, total cholesterol; HDL-C, high-density lipoprotein cholesterol; LDL-C, low-density lipoprotein cholesterol; TG, triglyceride; ApoA1, apolipoprotein A1; ApoB, apolipoprotein B; Lp(a), lipoprotein (a); HbA1c, hemoglobin A1c; Hcy, homocysteine.

### Comparisons of Metabolic Parameters Between Subjects With Hyperhomocysteinemia and Those With Normal Range of Homocysteine

As the elevated level of plasma Hcy is an independent predictor of cardiovascular disease (31), we divided the subjects into HHcy group (Hcy ≥ 15 μmol/L) and normal group (Hcy < 15 μmol/L) and compared the metabolic parameters. As shown in Table 2, of the 7,898 subjects, 32.3% (2,550) had HHcy. They presented higher levels of SBP, DBP, ALT, AST, ZJU index, Cr, urea, UA, LDL-C, non-HDL-C, TG, and ApoB and lower levels of eGFR, HDL-C, and ApoA1 than those with normal range of Hcy (all *p* < 0.001). HHcy was more common in males than in females. Higher percentage of subjects with HHcy developed hyperglycemia and hypertension than those with normal range of Hcy (all *p* < 0.001). As sex, age, and BMI, which are all important influencing factors for metabolic health, were different between the two groups, thus we further adjusted these factors. SBP, DBP, ALT, AST, ZJU index, Cr, UA, glucose, and HbA1c levels were still significantly higher in HHcy group after adjustment (all *p*^#^< 0.001). However, for the plasma lipid profiles, only HDL-C was significantly decreased in subjects with HHcy compared with those with normal range of Hcy after adjusting potential confounding factors (*p*^#^< 0.001).

**TABLE 2:** The comparison of clinical parameters between subjects with hyperhomocysteinemia (HHcy) and those with normal range of homocysteine (Hcy).

Parameters	Normal group (Hcy < 15 μmol/L)	HHcy group (Hcy ≥ 15 μmol/L)	*p*	*P* ^#^
N	5,348	2,550	N/A	N/A
Sex, male, *n* (%)	2,068 (38.7%)	2,086 (81.8)	<0.001	N/A
Age (years)	41.6 ± 12.6	42.7 ± 13.8	<0.001	N/A
BMI (kg/m^2^)	23.8 ± 3.7	25.4 ± 3.9	<0.001	N/A
Hcy (μmol/L)*	11.0 (10.0–13.0)	18.0 (16.0–24.0)	<0.001	<0.001
SBP (mmHg)	120.1 ± 17.2	126.4 ± 17.2	<0.001	<0.001
DBP (mmHg)	71.0 ± 11.3	75.6 ± 11.8	<0.001	<0.001
ALT (U/L)*	19.0 (14.0–26.0)	22.0 (16.0–32.0)	<0.001	<0.001
AST (U/L)*	21.0 (18.0–24.0)	22.0 (19.0–26.0)	<0.001	<0.001
ZJU	33.2 ± 5.4	35.4 ± 5.6	<0.001	<0.001
FIB4*	0.77 (0.57–1.09)	0.79 (0.56–1.16)	0.203	0.387
Cr (μmol/L)	70.0 ± 12.1	73.1 ± 13.1	<0.001	<0.001
Urea (mmol/L)	5.0 ± 1.2	5.3 ± 1.3	<0.001	0.354
eGFR [mL/(min⋅1.73 m^2^)]	113.2 ± 26.2	106.3 ± 27.3	<0.001	<0.001
UA (μmol/L)	330.0 ± 88.0	387.2 ± 92.5	<0.001	<0.001
TC (mmol/L)	4.94 ± 0.95	4.94 ± 0.94	0.842	0.259
HDL-C (mmol/L)	1.38 ± 0.36	1.21 ± 0.30	<0.001	<0.001
LDL-C (mmol/L)	2.96 ± 0.85	3.05 ± 0.85	<0.001	0.490
Non-HDL-C (mmol/L)	3.56 ± 0.93	3.72 ± 0.92	<0.001	0.996
TG (mmol/L)*	1.13 (0.81–1.67)	1.38 (0.96–2.02)	<0.001	0.520
ApoA1 (mmol/L)	1.42 ± 0.22	1.35 ± 0.21	<0.001	0.131
ApoB (mmol/L)	0.83 ± 0.21	0.86 ± 0.21	<0.001	0.881
Lp(a) (mg/dL)*	12.00 (7.00–24.10)	11.40 (6.50–22.70)	0.002	0.148
Glucose (mmol/L)*	4.81 (4.48–5.20)	4.83 (4.43–5.27)	0.636	< 0.001
HbA1c (%)*	5.40 (5.20–5.70)	5.50 (5.20–5.70)	0.063	< 0.001
Hyperglycemia, *n* (%)	656 (12.3%)	399 (15.6%)	<0.001	N/A
Hypertension, *n* (%)	763 (14.3%)	591 (23.3%)	<0.001	N/A

*Non-normally distributed continuous variables were compared after natural log-transformed. ^#^Adjusted for age, sex, and BMI. Continuous variables were analyzed using Student’s t-tests. Categorical parameters were compared with χ^2^-tests. A general linear model was used to compare data between groups after adjusting age, sex, and BMI. BMI, body mass index; SBP, systolic blood pressure; DBP, diastolic blood pressure; ALT, alanine aminotransferase; HHcy, hyperhomocysteinemia; Hcy, homocysteine; AST, aspartate aminotransferase; ZJU, Zhejiang University; FIB4, fibrosis-4; Cr, creatinine; eGFR, the estimated glomerular filtration rate; UA, uric acid; TC, total cholesterol; HDL-C, high-density lipoprotein cholesterol; LDL-C, low-density lipoprotein cholesterol; TG, triglyceride; ApoA1, apolipoprotein A1; ApoB, apolipoprotein B; Lp(a), lipoprotein (a); HbA1c, hemoglobin A1c.

### Plasma Homocysteine Was Associated With Lipid Profiles in This Adult Population

In light of the essential role of dyslipidemia in the development of cardiovascular disease, we further did a linear correlation analysis between the lnHcy level and plasma lipids. As shown in Table 3, before adjusting confounding factors, plasma Hcy levels were significantly positively correlated with LDL-C, non-HDL-C, TG, and ApoB and inversely related with HDL-C, ApoA1, and Lp(a) levels (all *p* < 0.001). Then, we adjusted the potential confounders after excluding multicollinearity variables. After adjusting age, sex, and BMI, there was still an inverse relationship between plasma Hcy and HDL-C (*p* < 0.001). Even after further adjusting SBP, DBP, ALT, AST, Cr, UA, and glucose, the negative association of Hcy with HDL-C (*p* < 0.001), ApoA1 (*p* = 0.035), and Lp(a) (*p* = 0.049) and the positive association with TG levels (*p* = 0.043) still existed.

**TABLE 3:** The association of lnHcy with plasma lipids.

Parameters	Model 1		Model 2		Model 3	
	β	*p*	β	*p*	β	*p*
TC (mmol/L)	–0.028	0.286	–0.045	0.122	–0.040	0.176
HDL-C (mmol/L)	–0.224	**<0.001**	–0.036	**<0.001**	–0.049	**<0.001**
LDL-C (mmol/L)	0.104	**<0.001**	–0.023	0.380	–0.024	0.368
Non-HDL-C (mmol/L)	0.196	**<0.001**	–0.009	0.736	0.009	0.753
TG (mmol/L)*	0.234	**<0.001**	0.007	0.634	0.031	**0.043**
ApoA1 (mmol/L)	–0.091	**<0.001**	–0.011	0.097	–0.014	**0.035**
ApoB (mmol/L)	0.043	**<0.001**	–0.002	0.690	–0.001	0.823
Lp(a) (mg/dL)*	–0.150	**<0.001**	–0.011	0.692	–0.058	**0.049**

Model 1, unadjusted; Model 2, adjusted for age, sex, and BMI; Model 3, adjusted for age, sex, BMI, SBP, DBP, ALT, AST, Cr, UA, and glucose. *Non-normally distributed continuous variables were analyzed after natural log-transformed. Data were analyzed using the linear regression models. P-values in bold are statistically significant. Hcy, homocysteine; TC, total cholesterol; HDL-C, high-density lipoprotein cholesterol; LDL-C, low-density lipoprotein cholesterol; TG, triglyceride; ApoA1, apolipoprotein A1; ApoB, apolipoprotein B; Lp(a), lipoprotein (a).

### Hyperhomocysteinemia Increased the Risk of Low High-Density Lipoprotein Cholesterol, in Which Apolipoprotein A1 Might Play a Role

Furthermore, we evaluated the risk of atherogenic dyslipidemia in subjects with HHcy. Compared with those with a normal range of plasma Hcy, the prevalence of low HDL-C, high LDL-C, and hypertriglyceridemia increased by 1.26 (*p* < 0.001), 0.18 (*p* = 0.002), and 0.76 times (*p* < 0.001), respectively, in individuals with HHcy before adjusting confounding factors. Nevertheless, only the association of HHcy with low HDL-C was still significant after adjusting age, sex, BMI, SBP, DBP, ALT, AST, Cr, UA, and glucose in the whole population [odds ratio (OR) 1.26; 95%CI (1.11–1.44); *p* < 0.001] (Table 4).

**TABLE 4:** Logistic analyses of the relationship between atherogenic dyslipidemia and HHcy.

Dependent variables	Model 1		Model 2		Model 3	
	OR (95%CI)	*p*	OR (95%CI)	*p*	OR (95%CI)	*p*
Hypercholesterolemia	1.00 (0.90–1.10)	0.719	0.93 (0.83–1.03)	0.167	0.93 (0.83–1.04)	0.211
Low HDL-C	2.26 (2.03–2.53)	**<0.001**	1.19 (1.05–1.35)	**0.006**	1.26 (1.11–1.44)	**<0.001**
High LDL-C	1.18 (1.06–1.30)	**0.002**	0.93 (0.83–1.04)	0.193	0.93 (0.83–1.05)	0.220
Hypertriglyceridemia	1.76 (1.59–1.95)	**<0.001**	1.01 (0.89–1.13)	0.925	1.06 (0.93–1.20)	0.392

*Model 1, unadjusted; Model 2, adjusted for age, sex, and BMI; Model 3, adjusted for age, sex, BMI, SBP, DBP, ALT, AST, Cr, UA, and glucose. Data were analyzed using the logistic regression models. P-values in bold are statistically significant. HHcy, hyperhomocysteinemia; Hcy, homocysteine; OR, odds ratio; CI, confidence interval; HDL-C, high-density lipoprotein cholesterol; LDL-C, low-density lipoprotein cholesterol.*

The relationship between HHcy and low HDL-C was inconsistent in different subgroups (Table 5). Significant associations were observed in subjects relatively old [OR 1.40; 95% CI (1.16–1.69); *p* < 0.001], both males [OR 1.23; 95% CI (1.07–1.41); *p* = 0.003] and females [OR 1.42; 95% CI (1.01–2.01); *p* = 0.046], both overweight or obese subjects and lean individuals [OR 1.27; 95%CI (1.08–1.49); *p* = 0.004 and OR 1.35; 95% CI (1.09–1.66); *p* = 0.006, respectively], and those without hyperglycemia [OR 1.27; 95% CI (1.10–1.47); *p* = 0.001] and hypertension [OR 1.34; 95% CI (1.16–1.55); *p* < 0.001] after adjusting other confounding factors. However, sex, age, BMI, and blood glucose level did not significantly affect the risk of low HDL-C in subjects with HHcy (*p* for interaction > 0.05). The low HDL-C risk was only significantly higher in those without hypertension than subjects with hypertension (*p* for interaction = 0.029).

**TABLE 5:** Risk of prevalent low HDL-C in different subgroups among subjects with HHcy compared with those with normal plasma Hcy.

Subgroups	OR (95% CI)	*p*	*p* for interaction	Cases/subjects
**Sex**				
Male	1.23 (1.07–1.41)	**0.003**	0.378	1,404/4,154
Female	1.42 (1.01–2.01)	**0.046**		292/3,744
**Age**				
≥42 years	1.40 (1.16–1.69)	**<0.001**	0.801	792/3,549
<42 years	1.16 (0.97–1.39)	0.112		904/4,349
**BMI**				
Overweight or obesity	1.27 (1.08–1.49)	**0.004**	0.180	1,148/3,230
Lean	1.35 (1.09–1.66)	**0.006**		548/4,668
**Hyperglycemia**				
Yes	1.17 (0.86–1.59)	0.315	0.472	352/1,055
No	1.27 (1.10–1.47)	**0.001**		1,344/6,843
**Hypertension**				
Yes	1.05 (0.80–1.37)	0.753	0.029	382/1,354
No	1.34 (1.16–1.55)	**<0.001**		1,314/6,544

*Data were analyzed using the logistic regression models after adjusting age, sex, BMI, SBP, DBP, ALT, AST, Cr, UA, and glucose. P-values in bold are statistically significant. OR, odds ratio; CI, confidence interval; HHcy, hyperhomocysteinemia; Hcy, homocysteine; HDL-C, high-density lipoprotein cholesterol; BMI, body mass index.*

It has been reported that Hcy could reduce the HDL-C level by suppressing the synthesis of ApoA1 in mice (12, 32). In this study, the Hcy level was inversely related with plasma ApoA1. Therefore, we further assessed the mediation role of ApoA1 in the effects of plasma Hcy on HDL-C in this population. As shown in Figures 1B,C, the total and direct effects of Hcy on HDL-C, as well as the total indirect effect *via* ApoA1, were all significant (all *p* < 0.001). The net mediation effects of ApoA1 before and after adjusting confounders were 46.9 and 30.6%, respectively.

### Remnant Cholesterol Was Dramatically Elevated in Subjects With Hyperhomocysteinemia and Had a Close Relationship With Other Plasma Lipids

**FIGURE 2:**
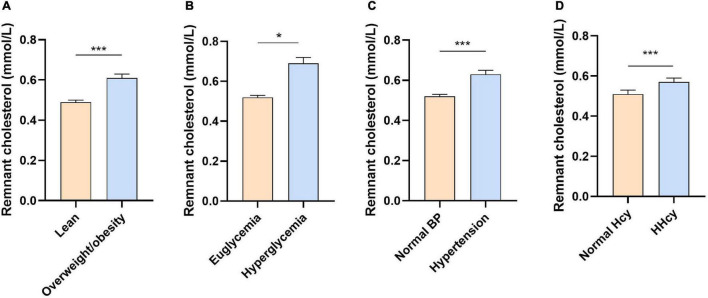
The concentration of remnant cholesterol in different groups. **(A)** Overweight or obesity vs. lean; **(B)** hyperglycemia vs. euglycemia; **(C)** hypertension vs. normal blood pressure; and **(D)** HHcy vs. normal Hcy. Data were presented as median ± 95% CI and were analyzed using Student’s *t*-test after natural log-transformed. *p < 0.05 and ***p < 0.001. BP, blood pressure; HHcy, hyperhomocysteinemia; Hcy, homocysteine.

Low-density lipoprotein cholesterol has been well-recognized as the atherogenic lipoprotein. However, no obvious correlation between HHcy and high LDL-C was detected in our study. Recently, emerging evidence implicated the critical role of TGRLs and their remnants (also named as RC) in ASCVD (19, 24). We assessed the level of RC in our study and showed that plasma RC was significantly increased in subjects with overweight and obesity, hyperglycemia, hypertension, and HHcy compared with their control groups, respectively (Figures 2A–D). Interestingly, the RC level was also dramatically increased in subjects with HHcy compared with those with normal range of Hcy after adjusting age, sex, BMI, SBP, DBP, ALT, AST, Cr, UA, and glucose (*p* = 0.025).

We further assessed the relationships between RC and other plasma lipids using the partial correlation coefficient analysis. As shown in Table 6, a significant positive association of RC with TG, TC, non-HDL-C, ApoA1, and ApoB and an inverse association with HDL-C, LDL-C, and Lp(a) were detected after adjusting confounding influencing factors (all *p* < 0.001, except ApoB *p* = 0.011).

**TABLE 6:** The relationship between lnRC and other plasma lipids after adjusting for age, sex, BMI, SBP, DBP, ALT, AST, Cr, UA, and glucose.

Parameters	r	*P*
TC (mmol/L)	0.166	**<0.001**
HDL-C (mmol/L)	–0.123	**<0.001**
LDL-C (mmol/L)	–0.230	**<0.001**
Non-HDL-C (mmol/L)	0.219	**<0.001**
TG (mmol/L)*	0.521	**<0.001**
ApoA1 (mmol/L)	0.127	**<0.001**
ApoB (mmol/L)	0.029	**0.011**
Lp(a) (mg/dL)*	–0.054	**<0.001**

*Non-normally distributed continuous variables were analyzed after natural log-transformed. Data were analyzed using the partial correlation coefficients. P-values in bold are statistically significant. RC, remnant cholesterol; TC, total cholesterol; HDL-C, high-density lipoprotein cholesterol; LDL-C, low-density lipoprotein cholesterol; TG, triglyceride; ApoA1, apolipoprotein A1; ApoB, apolipoprotein B; Lp(a), lipoprotein (a); BMI, body mass index; SBP, systolic blood pressure; DBP, diastolic blood pressure; ALT, alanine aminotransferase; AST, aspartate aminotransferase; Cr, creatinine; UA, uric acid.

### Homocysteine Was Independently Associated With Plasma Remnant Cholesterol Level Beyond Other Lipids

Finally, we analyzed the linear association of plasma Hcy with RC (Table 7) and found a significant positive correlation between Hcy and RC after adjusting age, sex, BMI, SBP, DBP, ALT, AST, Cr, UA, and glucose (β = 0.073, *p* = 0.004). This association was still significant even after adjusting the closely correlated plasma lipid profiles, including TG, LDL-C, HDL-C, ApoA1, ApoB, and Lp(a) (model 4–9), which indicated that plasma Hcy was independently related with RC beyond other lipids.

**TABLE 7:** The association of lnHcy with lnRC in different adjusting models.

Models	β	*p*
Model 1	0.153	**<0.001**
Model 2	0.071	**0.004**
Model 3	0.073	**0.004**
Model 4	0.054	**0.020**
Model 5	0.049	**0.032**
Model 6	0.048	**0.033**
Model 7	0.058	**0.008**
Model 8	0.052	**0.010**
Model 9	0.050	**0.012**

*Model 1, unadjusted; Model 2, adjusted for age, sex, and BMI; Model 3, adjusted for age, sex, BMI, SBP, DBP, ALT, AST, Cr, UA, and glucose. Model 4, model 3+TG. Model 5, model 4+HDL-C. Model 6, model 5+LDL-C. Model 7, model 4+ LDL-C+ApoA1. Model 8, Model 7+ApoB. Model 9, Model 8+Lp(a). Data were analyzed by the linear regression models. P-values in bold are statistically significant. Hcy, homocysteine; RC, remnant cholesterol; BMI, body mass index; SBP, systolic blood pressure; DBP, diastolic blood pressure; ALT, alanine aminotransferase; AST, aspartate aminotransferase; Cr, creatinine; UA, uric acid; TG, triglyceride; HDL-C, high-density lipoprotein cholesterol; TC, total cholesterol; LDL-C, low-density lipoprotein cholesterol; ApoA1, apolipoprotein A1; ApoB, apolipoprotein B; Lp(a), lipoprotein (a).*

## Discussion

In the large cross-sectional study among Chinese adults, we found that elevated Hcy levels were positively associated with plasma TG and inversely correlated with HDL-C, ApoA1, and Lp(a) after controlling age, sex, BMI, SBP, DBP, ALT, AST, Cr, UA, and glucose. HHcy significantly increased the risk of low HDL-C. More interestingly, Hcy was also independently correlated with plasma RC levels beyond other conventional lipids, including TG, LDL-C, HDL-C, ApoA1, ApoB, and Lp(a). Our findings suggest that identifying Hcy-related dyslipidemia risk, both traditional atherogenic lipids and RC residual risk, is clinically significant as we usher in a new era of targeted Hcy-lowering therapies to fight against dyslipidemia and ASCVD. Future studies are required to decipher the mechanism linking plasma Hcy and lipids, especially RC, independent of other traditional cardiovascular risk factors and to determine whether reducing Hcy improves lipids and clinical outcomes.

Plasma Hcy is an independent risk factor for ASCVD (33–36). Nevertheless, the mechanism linking Hcy to cardiovascular disease is not fully understood. Experimental studies indicated that HHcy-induced lipid alterations might play a role (11, 12). Barter et al. also proposed that HDL-C might be a link between Hcy and ASCVD (31). Although evidence is inconsistent and limited, previous epidemiological and clinical studies investigated the association of Hcy with plasma lipids. An Iran study carried out in patients with myocardial infarction first showed that Hcy was negatively correlated with HDL-C and positively related with LDL-C (37). Another study from India also demonstrated that plasma Hcy had an inverse relationship with HDL-C and a positive relationship with TG and very-low-density lipoprotein (VLDL) in subjects with coronary artery disease (38). Real et al. uncovered the inverse correlation between plasma Hcy and HDL-C in individuals with heterozygous familial hypercholesterolemia (39), which was confirmed by Guéant-Rodriguez et al. in the elderly population (40). Other studies also showed that HHcy could enhance LDL oxidation, which was modulated by the genetic and dietary factors (41, 42). In China, Huang et al. first explored the association of plasma Hcy with lipid profile in 192 hyperlipidemia patients and 208 normal individuals and found that HHcy was the risk factor for both hypercholesterolemia and hypertriglyceridemia (43). Then, Xiao et al. showed that plasma Hcy correlated inversely with ApoA1 and HDL-C among 2,058 patients undergoing coronary artery angiography (44). Subsequently, the positive correlation with TC, TG, and LDL-C and the inverse relationship with HDL-C of plasma Hcy were verified in Chinese patients who underwent physical examinations in Guangxi (45) and in a community-based population from Hunan (7). However, some studies did not support the correlation between Hcy and plasma lipids. Yadav et al. observed no significant correlation between plasma Hcy and TC, TG, and HDL-C in patients with ischemic heart disease (46). Another study found no significant association of Hcy with plasma lipids among 155 patients with diabetes (47). Lupton et al. suggested lower levels of LDL-C, non-HDL-C, and HDL-C, whereas higher levels of TG and VLDL-C in the fourth Hcy quartile among US adults before adjustment. Nevertheless, these associations disappeared after controlling for age, sex, HbA1c, insulin, Cr, and urea (15). In this study among Chinese adults aged 20–29 years in Beijing, we found that plasma Hcy levels were positively correlated with TG and inversely correlated with HDL-C, ApoA1, and Lp(a) after adjusting potential confounders including age, sex, BMI, SBP, DBP, ALT, AST, Cr, UA, and glucose. The prevalence of HHcy was 32.3%, which was lower than that of the study carried out in Guangxi (50.8%) (45). HHcy only significantly increased the risk of low HDL-C beyond the confounders. The different findings between this study and other studies might be attributable to the age, race, sex, diets, folic acid and vitamin B intake, geographic regions, physical activity, and sample number of the study population. Additionally, the indicators and confounding factors included in data analyses among the studies were also different. Our study included more plasma lipids, such as ApoA1, ApoB, and Lp(a), and controlled more influencing factors, such as glucose, blood pressure, and liver and kidney function, which were significantly elevated in subjects with HHcy in this study and were shown to be associated with Hcy levels in prior studies when performing the correlation analysis (15, 48, 49). In the future, more standard, large-scale, and prospective studies are needed to clarify the association of Hcy with plasma lipids.

Pharmacological LDL-C-lowering therapy *via* statin had obtained a significant impact on decreasing the incidence of ASCVD (50). However, distinct residual ASCVD risk has been reported among statin-treated patients, even with low levels of LDL-C (51). Low HDL-C was one of the most important factors for the residual risk, but there is no effective HDL-C-raising therapy till present (52). High Lp(a) was recently considered as a causal risk factor for ASCVD, whereas Lp(a) concentrations were reported to be primarily determined by genetics, and Lp(a)-lowering therapy is limited (53, 54). Although the residual risk may be attributable to many other factors, such as sedentary lifestyles, smoking, hyperglycemia, hypertension, and inflammation, the scientists have focused their interest in the TGRL or RC (16, 55, 56). The National Lipid Association writing group also recommended performing advanced lipid measurements, including RC, in these patients (57). TGRLs are highly heterogeneous lipoprotein particles derived from the intestine and liver and contain large amounts of TG and cholesterol, as well as transport approximately one-third of cholesterol in ApoB-containing lipoprotein particles (58). The cholesterol content of remnant lipoproteins was defined as RC and was highly atherogenic (59). When it comes to the association of Hcy with plasma RC levels, evidence is scarce. Only one study from the Very Large Database of Lipids in US adults showed that RC was 2–6% higher in the highest Hcy quartile before adjustment. When they controlled age, sex, HbA1c, insulin, Cr, and urea, the association disappeared (15). In this study, RC was defined as intermediate-density lipoprotein cholesterol (IDL-C) plus VLDL-C. In this study, RC was calculated as non-HDL-C minus LDL-C. Results showed that RC levels were significantly increased with Hcy elevation. After adjusting age, sex, BMI, SBP, DBP, ALT, AST, Cr, UA, and glucose, the positive association of Hcy with RC was still significant. The differences in populations, adjusted confounders, and the calculation method of RC might partially explain the inconsistency. Our study preliminarily indicated that HHcy-induced high level of RC might be an important mediating factor for the ASCVD risk.

A previous study demonstrated that the risk of myocardial infarction increased consecutively with an increase in RC even after adjusting LDL-C (60). Despite RC-related ASCVD risk proportional to the change of ApoB being reported (23), a recently published prevention study carried out in ASCVD-free US individuals showed that elevated plasma RC levels were associated with ASCVD independent of LDL-C and ApoB (24). In this study, the LDL-C was estimated by the Martin/Hopkins equation according to non-HDL-C and TG levels using an adjustable factor for the TG:VLDL ratio (61). These studies suggested that RC might modify ASCVD risk beyond the total particles. They also illustrated that there might be some indirect atherogenic mechanisms of RC, such as reflecting the increased activity of key lipid regulatory proteins, apolipoprotein C3 (ApoC3), or angiopoietin-like protein 3 (ANGPTL3). Consistently, our study showed that the association of plasma Hcy with RC levels was independent of other lipids, including TG, HDL-C, LDL-C, ApoA1, ApoB, and Lp(a), despite the estimation method of LDL-C is different from the study carried out by Quispe et al. (24). Mechanisms linking HHcy to high RC levels beyond the cholesterol, ApoA1, ApoB, and Lp(a) might exist and requires more investigation and verification.

The mechanisms underlying the association of plasma Hcy with lipid profile have been investigated in basic experimental studies. Liao et al. (12) and Mikael et al. (32) both showed that Hcy could inhibit the hepatic synthesis and expression of ApoA1, the main apolipoprotein of HDL-C, which explained not only the inverse association of Hcy with ApoA1 and HDL-C but also the Hcy-induced ASCVD risk. Our study similarly uncovered an inverse relationship between Hcy and plasma ApoA1 and HDL-C and showed that ApoA1 was an important mediator for the effects of plasma Hcy on HDL-C levels even after controlling potential confounders. In addition, it has been reported that Hcy could induce ER stress to activate sterol regulatory element binding proteins (SREBP), an important transcriptional factor involved in cholesterol and TG biosynthesis and thus increased cholesterol and TG levels (11). Moreover, the oxidative stress induced by protein misfolding in ER (62, 63) and global DNA hypomethylation modification (64) were both the potential mechanisms by which Hcy affected lipid metabolism. In the future, more studies need to be carried out to decipher the mechanism linking Hcy to atherogenic dyslipidemia, especially RC levels.

As published studies and our study both demonstrated that HHcy level increased the risks of dyslipidemia, how about decreasing the plasma Hcy? Recent small-scale clinical studies and animal experiments have preliminarily investigated the effects of folic acid supplementation, a well-acknowledged method to decrease Hcy, on lipid metabolism. Baszczuk et al. showed that 15 mg of folic acid per day for 45 days caused a significant growth of HDL-C and ApoA1 and a reduction of ApoB in 42 patients with primary hypertension (65). Vijayakumar et al. found that 400 μg of folic acid two times a day for 8 weeks significantly decreased LDL-C levels and the LDL-C/HDL-C ratio among 25 postmenopausal diabetic Korean women (66). The newest meta-analysis of 38 randomized controlled trials in adults summarized that folic acid supplementation decreased plasma TG and TC levels but did not affect LDL-C or HDL-C (67). Atherosclerotic and high-fat-diet-fed animal models also showed that folic acid increased HDL-C and enhanced antioxidative and anti-inflammation capacity (68, 69). Inconsistently, Satapathy et al. added folic acid and vitamin B12 for 8 weeks to 80 subjects with type 2 diabetes mellitus and did not find an improvement of lipid profiles (70). In the future, large-scale randomized controlled trials in different populations are required to clarify the beneficial effects of Hcy-lowering therapy on plasma lipids, particularly RC levels.

There are some limitations in our study. First, we did not collect covariates, such as dietary intake, vitamin B supplements intake, physical activity, and smoking and alcohol consumption information, which might affect the associations of Hcy with lipids. Second, the plasma levels of folic acid and vitamin B12 were not detected in our population, which was shown to regulate plasma Hcy levels. Third, RC was estimated by TC minus LDL-C and HDL-C but was not directly measured. Fourth, this is a single center study carried out in the Chinese population, thus cannot represent other regions and populations. In addition, this is a cross-sectional study and cannot uncover the causality between Hcy and lipids. Finally, the mechanism underlying the effects of plasma Hcy on lipid profiles is still largely unclear and needs more investigation.

## Conclusion

In conclusion, to the best of our knowledge, this is one of the largest studies to explore the association of plasma Hcy levels with conventional lipid profiles in China, and this is the first study to focus on the relationship between Hcy and RC levels in Chinese adults. Our study demonstrated that plasma Hcy levels were independently positively associated with TG and negatively associated with HDL-C, ApoA1, and Lp(a) after adjusting all potential confounders. HHcy significantly increased the risk of low HDL-C. The reduction of ApoA1 partially mediated this effect. More importantly, plasma Hcy was significantly associated with RC levels after adjusting confounding factors and even beyond other lipids, including TG, HDL-C, LDL-C, ApoA1, ApoB, and Lp(a). This study added Chinese evidence to the Hcy-induced dyslipidemia and provide novel insight for the prevention and treatment of atherogenic dyslipidemia or even ASCVD risk in patients with HHcy.

## Data Availability Statement

The original contributions presented in this study are included in the article/supplementary material, further inquiries can be directed to the corresponding author/s.

## Ethics Statement

The studies involving human participants were reviewed and approved by the Ethics Committee of the Beijing Chao-Yang Hospital affiliated with Capital Medical University. The patients/participants provided their written informed consent to participate in this study.

## Author Contributions

LZ and JL analyzed the data and drafted the manuscript. YA integrated the data. YW was responsible for recruiting subjects, collecting data, and revised the manuscript. GW contributed to the study design, data interpretation, and reviewed the manuscript. All authors read and approved the final version of this manuscript.

## Conflict of Interest

The authors declare that the research was conducted in the absence of any commercial or financial relationships that could be construed as a potential conflict of interest.

## Publisher’s Note

All claims expressed in this article are solely those of the authors and do not necessarily represent those of their affiliated organizations, or those of the publisher, the editors and the reviewers. Any product that may be evaluated in this article, or claim that may be made by its manufacturer, is not guaranteed or endorsed by the publisher.
